# Contributions to Operative and Mechanical Dentistry

**Published:** 1843-09

**Authors:** W. H. Elliot

**Affiliations:** Member of the American Society of Dental Surgeons.


### ARTICLE V.

*Contributions to Operative and Mechanical Dentistry.* By W. H. ELLIOT, Member of the American Society of Dental Surgeons. No. 2.

### ON EXTRACTING THE TEETH.

THIS important operation, at once the most simple, the most difficult, yet the most common, performed alike by the accomplished surgeon and the veriest quack, alternately baffling the efforts of the one and yielding to those of the other, is too often regarded with indifference, or looked upon as one of the evils attendant upon the practice of the profession. Nor can it be denied that even in the present age, either for the lack of suitable instruments, or the want of skill in the use of them, there exists, with some members of the profession, a strong aversion to it; for we not unfrequently meet with proof that their patients are sent away with a miserable palliative, or a mere apology for a remedy, when a single well directed effort would have rid them of a painful disease. He who is so weak as to shrink from the performance of this operation when the welfare of his patient demands it, is unfit to do it when it becomes precursory to others more lucrative yet less difficult; for in the most simple cases it requires the nicest skill and judgment, that he may give the patient the slightest possible pain, and that no avoidable injury be done to the surrounding parts.

Where then shall we look for a remedy? It can only be found in a thorough knowledge of the physiology and pathology of the dental organs, and in a multiplication of the simplest yet most effectual means, with a perfect knowledge of the use of them. The operator should have entire confidence in himself and in his instruments, so that he may be able to use them without hesitation, let the condition of the tooth be what it may. Under such circumstances he will do credit to himself and honour to the profession.

There is no operation in dental surgery that secures to the practitioner more effectually the full confidence of the patient, than the successful removal of a troublesome tooth.

There are few among us who are not aware that when it becomes necessary to extract a large number of decayed teeth or stumps, if the patient suffer the loss even of the most trivial by gentle means, he is sure to meet with no further opposition to his wishes; his gentlemanly deportment, his regard for the feelings of his patient, the delicate touch of the instrument, the ease with which he seems to perform the operation, and, finally, the successful removal of the tooth, with less than anticipated pain to the patient, all conspire to raise his fortitude to a confident degree, which places him unreservedly in the hands of the operator.

In the following remarks, I only wish to be understood as giving my own views, and not that the particular instruments and methods which I recommend are the only correct ones.

*The Key.*—Although I seldom use the key myself, and never except in those cases where the tooth has not sufficient strength on both sides to sustain the pressure of the forceps, it may not be out of place here, to make a few remarks on the best method of applying that instrument.

I would say, as a general thing, that all teeth should be turned towards the tongue; but this is sometimes impracticable. If the anterior portion of the tooth be too much weakened to bear the pressure of the hook, or if the space between it and the adjoining teeth be too narrow to allow its passage into the mouth, the other method may be adopted. The strength and position of the tooth, as well as the condition of the surrounding parts, should, before any particular method be decided upon, be well considered. This the operator must learn to do, if possible, at a single glance, lest a close and protracted examination alarm the patient.

There are several reasons why the fulcrum should not be placed on the outer gum. In the first place, the external gum being less often brought in contact with food or other foreign substances taken into the mouth, even when free from inflammation, is more highly sensitive to the touch of the instrument.

*Secondly.*—The internal alveolar plate of the lower jaw particularly, being thinner and consequently more elastic, will allow the tooth sufficient lateral motion to destroy its attachment on the opposite side, before the tooth begins to rise from the socket, and on that account it does not require half the vertical force to finish the operation that it would if the inner plate were stiff and unyielding, for in that case the whole periosteum must of necessity be torn asunder at the same instant. This remark applies more forcibly to the molares.

And lastly, the form especially of the lower teeth is more favourable to their internal exit.

One great reason why writers have generally recommended the application of the fulcrum to the outside of the lower jaw, is, because of its liability to slip when applied upon an internal gum which recedes.

For the benefit of those who still persist in the use of the key on all occasions, I will mention a slight improvement that I have made which secures the instrument effectually against the accident of slipping from its first position. This is the application of a second hook, to Dr. Parmly's improved key, the only one that can be used with any degree of safety. The improvement in question, is a broad thin hook fitted to the crowns of the teeth, having upon it a round screw shank, which passes through the head of the pivot, upon which the fulcrum turns. This hook when in use may hang upon the adjoining or second tooth removed on either side of the one to be extracted. By means of the screw on the shank, the fulcrum may be raised or lowered as occasion may require, in case of the absence of both teeth on either side, the hook may be drawn down by the same means, so as to rest firmly on the gum. This hook, instead of making the instrument more difficult of application, assists the operator very much in placing and holding it in a proper position until the first hook be adjusted.

The fulcrum should not, as has been recommended, be fixed upon a line with the edge of the gum, but rather be placed with so much of its face upon the gum, that it will not come in contact with the tooth. When it does come in contact with the tooth, the instrument at once loses a great portion of its power to elevate. It does so equally with or without the second hook.

That the advantage of having the fulcrum held firmly in its place is very great, is proven by the fact, that I have never in a single instance, to my knowledge, fractured the alveolus in the slightest degree, when the second hook had been used; indeed it would be almost impossible to carry away that portion of the alveolus which is firmly held down by the fulcrum.

The even and firm basis offered to the fulcrum by the internal gum of the upper jaw, needs no argument to establish its claim of superiority to that of the external.

I cannot agree with Dr. Shepherd in his opinion that he who has the ability to use the key successfully, ought not to change it for any other instrument; nor with Dr. Thackston, that the key should be abandoned entirely. The latter gentleman, in his letter* proves clearly enough that the fulcrum must rest upon the gum, in his supposed case, but unfortunately for his argument against the key, he stopped short of telling us how that operation might be performed with the forceps.

There is among several instruments that I have invented and constructed for the purpose of extracting teeth, one which I use with perfect success, and which I believe deserving of some consideration. It is a combination of the key and forceps; having upon it the beaks of forceps, and the handle of a common key instrument; and is so constructed that when power is applied to the handle, the beaks grasps the tooth with just sufficient force to prevent them from slipping, be that power much or little. The position of the handle of this instrument is always such that the operator is able to use his whole force upon it without the least inconvenience. Its lateral force is equal to that of the key, and its vertical, equals that of the forceps.

*The Forceps.*—The next instrument that seems to claim our attention is the forceps.

The first object to be secured after fitting accurately the beaks of the forceps to the necks of the teeth, for which they are intended, is to bend the handles into such a form that during the operation, the operator's hand, arm and chest, may be in that position in which he can exert his utmost strength if occasion require without the least awkwardness or inconvenience.

In the removal of all teeth with the exception of those having roots that admit of a rotary movement, the instrument should have two lateral motions, first inward and then outward, and that

* Journal, page 185, vol. 1st.

with an effort so determined, that were its attachment twice as strong as it usually is, the operation would be performed in precisely the same length of time. These motions should not be made so rapidly as to prevent the operator from observing with precision the result of each effort to loosen the tooth, or cause him to forget that it is always liable to crush under the pressure of the forceps. In case one side of a tooth be very much weakened by decay, the instrument should have but one lateral motion, and that towards the weaker side.

The vertical force should always commence with the lateral, and increase until the tooth leaves the jaw; if on the contrary the tooth be carried from side to side before the vertical force be applied to it, all that pain which is occasioned by forcing one prong into the jaw, is unnecessary, for although the periosteum may be partially crushed, its attachment to the jaw is not the less strong.

The strongest of all forms that forceps possibly can take is that of the hawk's-bill, therefore just in proportion as they approach this form, they may be made lighter and stronger, it is on that account for extracting the lower molares that I prefer the forceps as improved by Dr. Flagg to those recommended by Mr. Snell. Besides, the position of the hand while operating with Flagg's forceps is much more natural. For extracting the lower dentes sapientiæ and bicuspides, I use the same form of instrument with the exception that the beaks of those for drawing the posterior teeth have a little more curvature, and those for the anterior a little less.

In removing teeth from the right side of the lower jaw, the operator's first finger and thumb should be next to the joints of the instrument, but in drawing them from the left side, the little finger should be next to the joints.

Whether operating upon the right or left side of the jaw, the position of the operator should be the same, that is, with the head of the patient resting against his left breast, and supported in its position by his left arm, the chin being secured by his left hand.

For extracting the molares, dentes sapientiæ and bicuspides from the upper jaw, I use instruments differing in their form from any that I have ever seen in use by others. This difference consists in giving to the instruments recommended by Mr. Snell, a bend between the handles and joints, exactly opposite to that between the joints and beaks, so that those for drawing the dentes sapientiæ, present two angles of about forty-five degrees each, one at each extremity of the joints; those intended for the molares are bent to angles of about thirty-five degrees, and those for bicuspides to angles of about twenty-five.

Owing to the handles and beaks being bent in different directions, the instrument is as well balanced, as if it had no angles whatever in it. A line drawn from the points of the beaks to the extremity of the handles, passes through the centre of the joint. This form of instrument enables the operator to extract a dens sapientiæ with the same facility and in the same manner, as if it were situated in the anterior part of the mouth.

As the force is applied longitudinally with the handles, one of them should be bent in the form of a hook, to prevent the hand from slipping.

*The Screw.*—The screw when properly made and skilfully managed, is of all instruments the least productive of pain.

Every dentist should supply himself with screws of different sizes, each screw being accompanied with a tap and drill. The tap and screw should, like the drill, be a separate piece from the rest of the instrument, so that others differing in size and length, may be put into the same handle. The threads should be fine, and formed like those of a common wood-screw, thin and sharp with a broad and deep space between them.

The screw and tap should be made exactly alike, with the exception that the tap should have deep longitudinal grooves filed upon it. These grooves should be cut deeper than the threads so that the tap may serve as a broach, at the same time that it cuts the thread in the root.

The results of numerous experiments upon the screw, have led me to believe that its success, almost entirely depends upon its form, and upon the form of the hole in the root into which the screw is inserted.

The form of the screw should be slightly tapering, equal to an angle of five degrees; when made to taper more, it requires too much force to give the screw a hold upon the tooth; when made to taper less, the tap cannot be withdrawn, without doing more or less injury to the thread which it has cut in the root.

The hole in the fang should have the same diameter within as at its orifice, leaving it to the tap to give it the proper form to receive the screw.

For the roots of posterior teeth, the drilling instrument should have upon it an universal joint, with a support to hold it at any required angle. For tapping and extracting such stumps, the instruments into which the taps and screws are placed for use, should like the drilling instrument, have an universal joint upon it, of sufficient strength to raise the fang from the jaw, it should also have a movable fulcrum so arranged, as to be placed on the top of any of the neighbouring teeth. This fulcrum of course cannot be used in the absence of the joint, nor is it required, except when the position of the tooth makes the joint equally necessary.

The teeth upon which the fulcrum rests, should always be protected by a slip of soft pine wood.

I sometimes extract the root of the upper front teeth by striking them out at a single blow. For this purpose I attach to the screw a rod about sixteen inches in length, made of steel wire, upon which I place a leaden weight of about three pounds, then when the screw is firmly fixed in the root, I slide the weight against a button on the end of the rod, with sufficient force to effect the object.

I have been positively assured by persons for whom I have drawn teeth in this manner, that the operation gave them no pain whatever.*

* The writer thinks that his first article has not been *fairly* treated in the appended note.

In regard to the originality of his views as to the form the cavity should take, *par preference,* "smaller within than at the orifice," he believes he stands unsupported by a single previous writer.

The annotator says—"there are few dentists, who have much experience, who are not aware of the necessity of making the cavity the same or nearly the same size at the orifice as at the bottom," and refers to Bell and Harris, also Parmly's introductory address, in No. 1, vol. iii.

What the writer contends is, that to him belongs the *novelty* of making in the generality of cases, the cavities of a shape *exactly reversed* of those recommended by most of the best and later writers on operations on the teeth, and subjoins extracts bearing him out from the writers quoted by the annotator.* By reference to which it will be seen by every unprejudiced eye, that those dentists of experience do not, as is misstated by the annotator, make the cavities the same or nearly the same size at the orifice as at the bottom;" but recommend them to be *larger* as stated by the writer.

In regard to the opinions of "dentists who have much experience," for them he entertains a becoming respect; but regarding error as the result of "*experientia fallax*" and believing that "*experientia staltorum magistra,*" he cannot yield up a tested proof nearly because it conflicts with preconceived notions, or wars against prevalent practices. There were few philosophers of experience before the days of Galileo, but what regarded the earth as the centre around which the sun revolved; yet the Copernican system has proved the only true one, the opinion of the papal court to the contrary notwithstanding, nor have we many enlightened dentists at the present time who would advocate their own rude mechanical contrivances of sea-horse plates, animal teeth, &c. in use *a dozen years ago,* merely because they were sanctioned by experience. Such a praxis of reasoning is as puerile as illogical.

In his numbers the writer shall advance nothing that will not stand the test of proof; ond whilst he invites criticism, he asks in all fairness, that it will be conducted in a spirit of inquiry, and that his own views may not be misstated and the writings of others opposed to them misquoted, and be made to say what they have not charged themselves with in print.

It becomes not the writer to speak immodestly of his own experience in dental surgery, but he may be permitted to say, if an extensive practice can add ought to his knowledge, he is fortunate in having such a one. His object in writing these numbers is not so much to extend his own fame, as to make known in liberality and good feeling towards his professional brethren, what he deems to be improvements in dental practice, and the manufacture of instruments; yet he may be indulged in a laudable ambition to improve his profession without subjecting himself to unfair statements of his views, and misapprehension of his intentions.

*"The cavity to be filled should be rather narrow in proportion to its depth, and *if practicable*, should be made rather larger within than at its external orifice."—BELL, 142.

"The bottom of the cavity should always be a *little larger* than the top, or at *least as large*."—HARRIS, 157.

"It is essential in all cases that the cavity which receives the gold should be made *larger within*, than at the orifice or external opening."—PARMLY's introductory address, No. 1, vol. iii.

---

The injustice of which Mr. Elliot complains as having been done him by the author of the note to his former communication, in the expression of the belief that he was in error with regard to "the originality of his views, on the best form the cavity" in a tooth, preparatory to filling, should have, we are certain was not intended. The writer of the note in question, says—

"There are few dentists who have much experience, who are not aware of the necessity of making the cavity the same or *nearly* the same size at the orifice as at the bottom;" while Mr. E. states, "that in, by far the greater number of cases, they (the cavity) should be made quite as large in circumference at the orifice, as at any other part, and in very many cases larger." The views of the authors referred to by the former, as may be seen by the quotations made from them by the latter, we think, sustains the opinion expressed by him upon the subject. The only material difference of opinion then between the writer of the note and Mr. E. is, that the latter claims to be the first to have recommended that the cavity in a tooth to be filled, should never be larger interiorily than it is at the opening, and that in very many cases it should not be as large; while the former contends that the majority of experienced practitioners would have the orifice and bottom of the cavity of the "same or *nearly* of the same size."

As to the supposable advantage for which Mr. Elliot contends, of having the cavity in any case larger at the opening than within, we differ with him in opinion. If the gold, or material employed, whatever it may be, for filling the tooth, be packed with no other instruments than the common round-pointed pluggers, it could be more thoroughly done, we admit, in a cavity thus shaped, than in one as large or larger at the bottom than at the orifice; but if properly constructed wedge-pointed pluggers be used, and they are the only instruments by which a filling can be thoroughly condensed, it may be more firmly packed in a cavity as large or even a little larger within than at the opening, than in one of a reversed shape, for the plain and simple reason, that in the latter case, the pressure employed to force the foil out against the walls of the cavity, would displace it from the tooth, and thus defeat the object intended to be accomplished.—*Balt. Ed.*]

